# Supplementary figures and images for: Sex identification in embryos and adults of Darwin’s finches
Source: PLoS One. 2021 Mar 5;16(3):e0237687. doi: 10.1371/journal.pone.0237687 (PMC7935298; doi:10.1371/journal.pone.0237687)

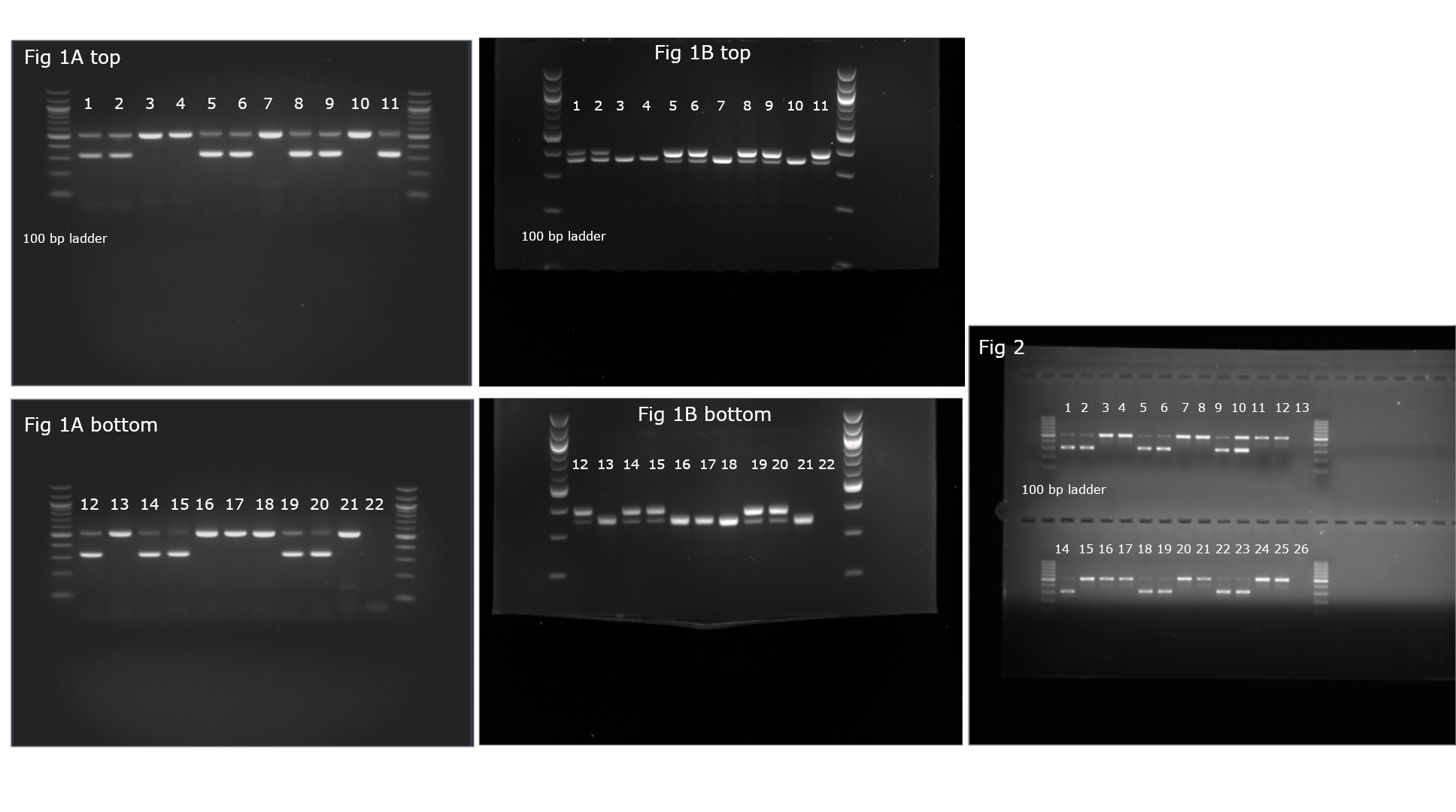

Supplement: S1 Raw images — (TIFF) [file pone.0237687.s001.tiff]
